# The Influence of Typhoid Fever upon Insanity

**Published:** 1888-01

**Authors:** 


### *THE INFLUENCE OF TYPHOID FEVER UPON INSANITY.*

"It is an ill wind that blows no one good." The appearance of typhoid fever among the patients at the State Hospital for the Insane, at Norristown, Pa., has given rise to a great deal of anxiety; but it has had a most fortunate and curious result in curing some of the patients, who contracted the fever, of the insanity. One woman, about fifty years old, who had suffered with chronic melancholia for several years, has been discharged perfectly well. Another patient, about twenty years of age, who had been in the hospital eight months with a second attack of acute mania, though subsiding somewhat at the time of the invasion, has gone home perfectly well. Another patient, a girl about eighteen years old, was beginning to recover from acute mania, when she sickened with typhoid fever. After the fever left her, she seemed quite bright, although she is now inclined to be very dull and stupid. At any rate, a decided change has been produced in the mental condition of this patient.

It is well known that the bodily conditions are frequently altered after typhoid fever; the lean may grow corpulent, and those who were previously fat, may grow lean. But it is not so generally known that disturbance of the cerebral functions are favorably affected, or that even the moral principles of an individual may undergo a decided change. After this fever, some persons have been known to develop a propensity for stealing. Dr. Crothers reports a case of a student of theology who, on recovering from typhoid, "betook himself to such a life as compelled him to pass most of his time in jail."

Dr. W. F. Waugh reported similar cases to the Pennsylvania State Medical Society in 1886. One young man, after being ill with typhoid fever for thirteen weeks, exhibited the delusion of grandeur, and developed most unnatural tastes, acting consistently the part of the wealthy youth who is sowing his wild oats. A more gratifying phase of his mental revolution was the development of a talent for the work of a solicitor, which he never exhibited before his illness.

These curious results indicate the profound influence which such a disease as typhoid fever may exert upon the human economy, and discover it in an unexpected *role* of beneficence. In such cases as we have referred to, it has proven a blessing, although a blessing very well disguised.—*Medical and Surgical Reporter.*

